# Anti-glomerular basement membrane disease with rupture of the newly formed bilateral corpus luteum cysts: A case report

**DOI:** 10.1097/MD.0000000000031643

**Published:** 2022-10-28

**Authors:** Pei-Yi Luo, Xia Chen, Lu Cheng, Liang Ma, Shen-Ju Gou

**Affiliations:** a Department of Nephrology, West China Hospital of Sichuan University, Chengdu, China; b Kidney Research Institute, West China Hospital of Sichuan University, Chengdu, China.

**Keywords:** anti-glomerular basement membrane (anti-GBM) disease, case report, corpus luteum cysts, pregnancy

## Abstract

Anti-glomerular basement membrane (anti-GBM) disease during gestation is sparse and even rarer when combined with bilateral large corpus luteum cysts. In this case, we report a case of anti-GBM disease in the early stage of pregnancy with ruptured newly formed bilateral large corpus luteum cysts.

**Patient concerns:**

A 24-year-old female was initially diagnosed with anti-GBM disease. During treatment, abdominal distention and vaginal bleeding successively staged. The results of the first gynecological ultrasound and abdominal CT were negative.

**Diagnosis:**

Based on the dynamic imaging change of the ovaries, the elevated human chorionic gonadotropin (hCG) and sex hormones, and the pathological findings, a diagnosis of anti-GBM disease with rupture of the newly formed bilateral corpus luteum cysts during early pregnancy was considered.

**Interventions:**

The patient was treated with corticosteroids, plasma-exchange along with intensive hemodialysis. Then, to confirm the diagnosis, laparoscopic debulking of bilateral ovarian cysts and curettage were performed.

**Outcomes:**

After treatment, the anti-GBM antibody titer declined and the condition of the patient was still stable 2 months following discharge.

**Lessons:**

As clinicians, we should be aware that even if the first imaging tests are negative, the relevant indicators should be reviewed dynamically based on the condition of the patients. Additionally, this case raised the question of whether anti-GBM disease was associated with pregnancy and giant corpus luteum cysts, which needs further investigations.

## 1. Introduction

Anti-glomerular basement membrane (anti-GBM) disease is a sparse and fatal autoimmune disease, characterized by anti-GBM autoantibodies deposition along the basement membrane of the glomeruli. According to the Chapel Hill consensus conference nomenclature, it is a small-vessel vasculitis affecting glomerular capillaries, pulmonary capillaries, or both.^[1]^ The incidence of anti-GBM disease is less than 1 case per million people,^[2]^ predominantly affecting the 20 to 30 and 60 to 70 age groups.^[3]^ The diagnosis of anti-GBM counts on the detection of anti-GBM antibodies. Associated with renal and/or pulmonary manifestation such as rapidly progressive glomerulonephritis and/or alveolar hemorrhage.^[4]^ For the initial management a combination of corticosteroids, plasma-exchange, and immunosuppressants is recommended. Despite aggressive treatment, patients with anti-GBM disease still face a poor prognosis, consequently leading to end stage renal disease and, even death.

The etiology of anti-GBM disease remains unclear. However, influenza virus, smoking, inhalation of hydrocarbons, and other environmental triggers might be involved in the development of anti-GBM disease.^[5–8]^ To the best of our knowledge, anti-GBM disease was rarely complicated with gestation. Here we report a case of anti-GBM disease in the early stage of pregnancy with ruptured newly formed bilateral large corpus luteum cysts.

## 2. Case report

A 24-year-old female (height: 153 cm, body weight: 43 kg) was admitted to a local hospital on 3rd April 2021 with a 4-day history of vomiting, oliguria, gross hematuria, cough, expectoration, and dizziness. The serum creatinine was 1568 μmol/L (17.7 mg/dL) and anti-GBM antibody was positive. The patient received hemodialysis treatment. On April 13th, 2021, the patient was transferred to our hospital.

In her medical history, a miscarriage in 2019 was recorded. Additionally, the patient reported menstruation twice in March and sexual intercourse in late March or early April. Last vaginal bleeding had initiated on April 4th, 2021 and lasted for 2 to 3 days, with less volume by comparing to previous menstrual periods. No progestin or estrogen was used before.

On admission, the results of routine peripheral blood tests were 55 g/L for hemoglobin, 10.62 × 109/L for leukocyte count, and 88 × 109/L for platelet count. Protein-creatinine ratio in urine was 0.167 g/mmol. The fecal occult blood test was negative. The blood biochemistry analysis revealed the following: albumin, 28.9 g/L; globulin, 23.1 g/L; alanine Aminotransferase, 20 IU/L; total bilirubin, 12.4 μmol/L; blood urea nitrogen, 9.8 mmol/L; serum creatinine, 438 μmol/L (4.95 mg/dL); uric acid, 136 μmol/L; C-reactive protein, 2.55 mg/L and procalcitonin, 0.50 ng/mL. Clinical immunology tests revealed the following: IgG, 20.4 g/L; serum IgG4, 0.342 g/L; IgM, 1230 mg/L; IgA, 2780 mg/L; C3 complement, 0.7590 g/L; C4 complement, 0.2260 g/L. The direct Coomb’s test was positive, whereas the retest was negative. Anti-GBM antibodies were detected in serum (with a tier > 500 AU/mL). Anti-nuclear antibody was dubious. The anti-neutrophil cytoplasmic antibodies, anti-cardiolipin antibodies, anti-beta2 glycoprotein, and lupus anticoagulant were negative. The tests targeting the hepatitis B, hepatitis C, HIV, and syphilis markers were all negative. A serum immunofixation electrophoresis revealed no monoclonal proteins. Transabdominal ultrasound revealed kidney sizes of 12.6 cm × 5.3 cm × 4.4 cm and 12.6 cm × 5.7 cm × 5.3 cm. Computed tomography (CT) scan revealed the presence of bilateral small pleural effusion and signs of peritonitis.

**Figure 1.**
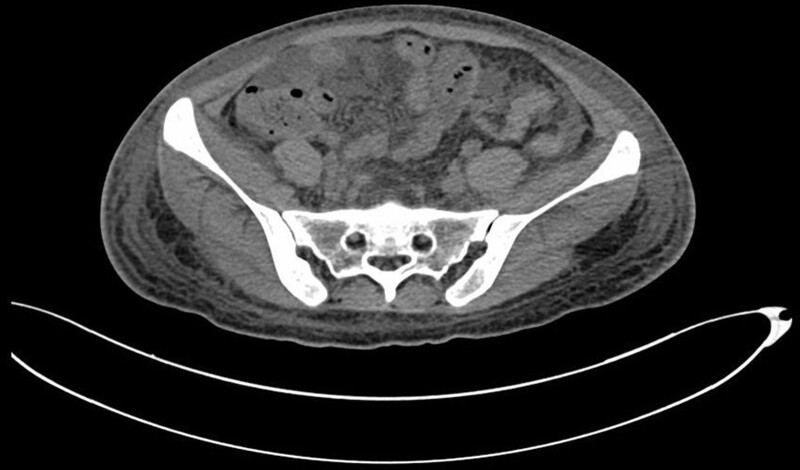
Abdominal computed tomography on the 9th day of admission showing no abnormalities in the uterus and bilateral adnexal areas.

**Figure 2.**
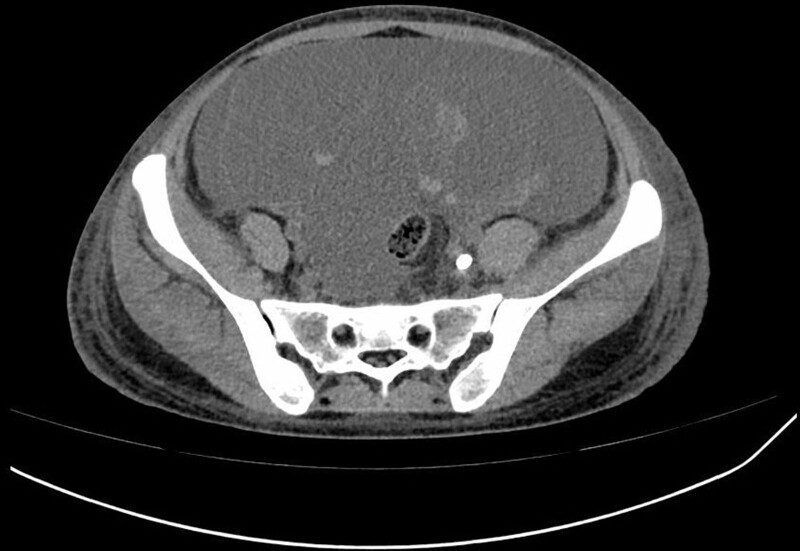
Abdominal computed tomography on the 40th day of admission showing multi-room cysts in the pelvis which were close with bilateral adnexal areas.

**Figure 3.**
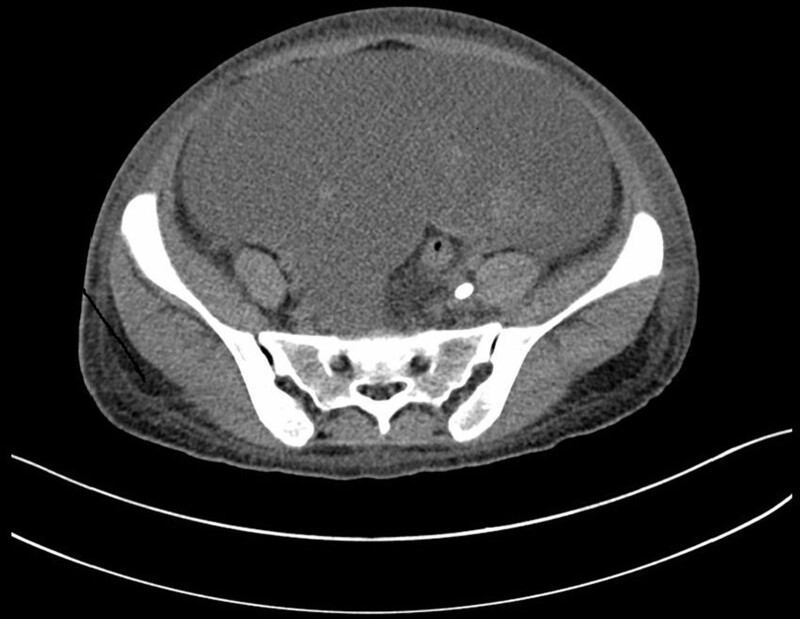
Abdominal and pelvic computed tomography angiography showing no obvious signs of vascular hemorrhage.

The patient was diagnosed with rapidly progressive glomerulonephritis due to anti-GBM glomerulonephritis. Intensive hemodialysis, plasma-exchange, and methylprednisolone of 40 mg/day were started immediately. On the 9th day of admission, new symptoms including bloating, diarrhea, and a tiny quantity of vaginal bleeding emerged. However, further gynecological ultrasound and abdominal CT identified no abnormalities in the uterus and bilateral adnexal areas (Fig. 1). On the same day, the vaginal bleeding ceased spontaneously without special medical intervention. On the 19th day of admission, hemoptysis appeared and aggravated gradually. Three days of methylprednisolone pulse (200 mg/day for 2 days and 500 mg for 1 day) were initiated and antibiotic medications were used. After these interventions, hemoptysis was gradually alleviated. However, on the 39th day of admission, abdominal distention reappeared without vaginal bleeding. The abdominal CT showed massive fluid accumulated in the abdominopelvic cavity and multi-room cysts in the pelvis which were closed with bilateral adnexal areas. The size of the largest cyst was about 10.0 cm × 10.1 cm (Fig. 2). The hemoglobin of the patient dropped from 84 g/L to 64 g/L during 3 days. Meanwhile, an abdominocentesis was performed and finally confirmed as hemoperitoneum. To further confirm the bleeding site, we then performed abdominal and pelvic CT angiography. No signs of vessel hemorrhage were founded (Fig. 3). The serum human chorionic gonadotropin (hCG) was detected, and the level was 169.59 mIU/mL (reference, < 3.81 mIU/mL). The level of estradiol was 1689 pg/mL (reference, first trimester 154–3243 pg/mL, second trimester 1561–21280 pg/mL, third trimester > 8525 pg/mL) and the level of progesterone was 14.20 ng/mL (reference, first trimester 11.0–44.3 ng/mL, second trimester 25.4–83.3 ng/mL, third trimester 58.7–214.0 ng/mL). A repeated transvaginal ultrasonographic examination was performed and revealed several cystic occupancies with blood flow signals in the cysts’ wall in the 2 enlarged ovaries. An endometrial thickness of 1.5 mm. No gestational sac within the uterine cavity was detected. Besides, fluid with echoes compatible with blood as deep as 4.5 cm was noted in the pelvic cavity. Combined with the reports of abdominal CT, gynecologic ultrasound, and the dropping level of hemoglobin, it was speculated that the patient had bleeding from a ruptured cyst in the adnexal region.

**Figure 4.**
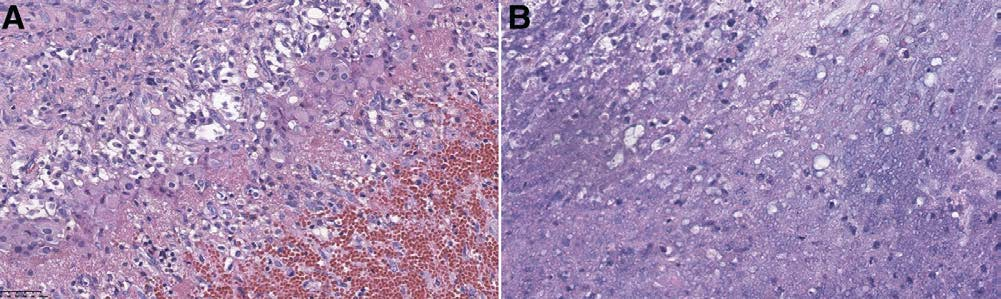
The histopathological findings of the surgical specimen. (A) Hematoxylin and eosin–stained section of the ovary showing luteal cysts with hemorrhage (amplification × 400). (B) Hematoxylin and eosin–stained section of the pathological tissue after curettage showing endometrium with focal inflammatory exudative necrosis and decidualization, and endometrium with secretory phase tissue (amplification × 400).

On the 42nd day of admission, the patient received the laparoscopic exploration and exfoliation of the bilateral ovarian cysts and curettage. During laparoscopy, hemoperitoneum and pelvic hemorrhage with approximately 3000 mL were observed. The cystic occupations were in the form of partitions in the bilateral ovaries with clear yellowish fluid inside. There was no bleeding at the fimbria of fallopian tubes and no purple-blue nodules or enlargements on the surface of either fallopian tube. The diagnosis of bleeding from ruptured bilateral adnexal cysts was established and the suspected diagnosis of spontaneous abortion was made. Postoperative pathology revealed luteal cysts with hemorrhage (Fig. 4A), endometrium with focal inflammatory exudative necrosis and decidualization, and endometrium with secretory phase tissue (Fig. 4B). After surgery, the patient’s abdominal distension disappeared and hCG gradually declined to 23.25 mIU/mL on the 6th postoperative day and return to normal with a level of 2.79 mIU/mL on the 16th postoperative day.

After applying plasma-exchange for 21 times, intensive corticosteroids, and anti-infective therapy, the patient’s hemoptysis was alleviated. The anti-GBM antibody titer dropped to 37.2 AU/mL. The general condition of the patient was stable when we followed up at 2 months after discharge, but the patient was still dialysis-dependent because of anuria.

## 3. Discussion

Based on the dynamic imaging change of the ovaries, the elevated hCG and sex hormones, and the pathological findings, early pregnancy with rupture of the newly formed bilateral corpus luteum cysts was determined in the present patient with anti-GBM glomerulonephritis. To date, anti-GBM disease occurring during gestation had only been reported in a few cases.^[9–18]^ Patients with newly formed bilateral large corpus luteum cysts in patients with anti-GBM disease and pregnancy were not reported.

In the present case, the vaginal bleeding has appeared at the beginning of the admission, the negative gynecological ultrasound and abdominal CT and the use of anticoagulation in dialysis distracted the doctors’ attention from the possibility of pregnancy in the early stage. The early pregnancy was lately confirmed by the elevated hCG and progesterone, as well as the surgical pathology. The pathology of endometrium was endometrial decidualization. Generally, endometrial decidualizes throughout the secretory phase of the menstrual cycle, which lasts until blastocyst implantation.^[19]^ During pregnancy, decidualization primarily functions to govern conceptus implantation and facilitate the harmonic growth of the embryo and its annexes.^[20]^ Hence, decidualization of uterine tissue was essential to establish pregnancy.^[21]^ By reversely thinking of the present case, the presence of vomiting might be the early reaction of pregnancy or the gastrointestinal symptoms of azotemia. The severe renal dysfunction from anti-GBM disease also contributed to the ignorance of the possibility of pregnancy. The present case revealed the importance of women’s menstrual history and sexual intercourse history inquiries, even in seriously ill patients. If the patient’s description was not clear, further investigation by both hormone testing and imaging tests should be conducted.

In this case, anti-GBM disease occurred in the early stage of the pregnancy. This condition raised the question of whether there was any association between the pregnancy and anti-GBM disease. Kai et al^[16]^ reported a case of a 28-year-old female who was diagnosed with anti-GBM glomerulonephritis during the first trimester of pregnancy. The edema occurred as early as the fifth week. In addition, Kai et al summarized 9 cases of anti-GBM disease occurring during pregnancy, most of which were diagnosed in the first or second trimester of pregnancy. Since the anti-GBM disease was rare and the cause was not clear yet, the reported case here and previous ones suggested an association between pregnancy and anti-GBM disease, which needs further investigation.

According to the previous case, the placenta may exhibit a potential role in the amelioration of anti-GBM disease. Deubner et al^[10]^reported 1 case of a 21-year-old female who had glomerulonephritis with anti-GBM antibody during pregnancy. The patient’s renal function declined slowly during pregnancy. However, it has deteriorated rapidly after delivery. The immunomodulatory milieu during pregnancy may account for the slowly declined renal function. The placenta may serve as an adsorbent by providing abundant basement membrane surfaces for binding the circulating anti-GBM antibodies. The decreased circulating anti-basement membrane antibodies bring less harms to the fetus and the pregnant. As opposed to the report from Deubner et al, Nair et al^[11]^ reported a 23-year-old female who was diagnosed with Goodpasture’s syndrome during the thirteenth week of pregnancy. The patient developed severe renal failure and had high anti-GBM antibody titers. Despite aggressive treatments were used, the patient remained oliguria and dialysis-dependent. But her renal functions normalized and the anti-GBM antibody titer became negative after the pregnancy was terminated. In our case, the patient’s renal function did not recover after the surgery. As indicated by the inconsistent renal function outcomes in these cases, the protective role of the placenta was doubtful.

Of note, in this case, it was sparse that the patient not only had anti-GBM disease during pregnancy but also developed bilateral large corpus luteum cysts of more than 10 cm in a short time, which then ruptured and hemorrhaged. The corpus luteum cysts are mostly functional ovarian cysts, which develop during the second phase of the ovarian cycle.^[22]^ The functional ovarian cysts are the most common ovarian masses of reproductive age and are influenced by the ovarian cycle and hormonal changes.^[23,24]^ Most corpus luteum cysts are asymptomatic and will resolve spontaneously in the absence of pregnancy or after the first trimester of pregnancy, or after placental maturation.^[22]^ However, it is still possible for the corpus luteum cysts to rupture or twist, causing acute abdominal pain and pelvic bleeding.^[25]^ Ruptured corpus luteum cysts in early pregnancy are often misdiagnosed as an ectopic pregnancy. In our case, the pregnancy test of the patient was positive, but the ultrasound examination showed no intrauterine gestational sac. The patient was likely to have ectopic pregnancy. However, the laparoscopic surgery denied this possibility. The undetected gestational sac in the uterus may be attributed to the pregnancy that is too early to visualize or spontaneous abortion.

The etiology of functional ovarian cysts is still unknown. Tamoxifen, immunosuppression, smoking, and marijuana smoking are common risk factors for functional ovarian cysts. Higher body mass index and estrogen contraceptives can reduce the risk of functional cysts.^[26,27]^ Generally, the diameters of corpus luteum cysts during menstruation or pregnancy are less than 2 cm. Hallatt et al^[28]^ reported that only 12% of 173 patients with corpus luteum rupture had corpus luteum cysts > 5 cm. Meanwhile, 66% rupture occurs in the right ovary, while 32% rupture in the left ovary. In the present case, bilateral corpus luteum cysts formed in a short time, with sizes of both sides larger than 10 cm. According to the previous description, the growth of functional ovarian cysts might be immune-related. The coexistence of anti-GBM disease was an autoimmune disease. Whether bilateral large corpus luteum cysts were related to anti-GBM disease remains unclear, further investigation was needed.

In conclusion, as clinicians, we should be aware that even if the first imaging tests are negative, the relevant indicators should be reviewed dynamically based on the condition of the patients. Additionally, this case raised the question of whether anti-GBM disease was associated with pregnancy and giant corpus luteum cysts, which needs further investigations.

## Acknowledgments

We thank the patient and clinical stuffs involved in her therapy for their medical and spiritual support.

## Author contributions

**Conceptualization:** Pei-Yi Luo.

**Data curation:** Pei-Yi Luo, Xia Chen, Lu Cheng.

**Methodology:** Lu Cheng, Liang Ma, Shen-Ju Gou.

**Writing – original draft:** Pei-Yi Luo, Xia Chen.

**Writing – review & editing:** Pei-Yi Luo, Liang Ma, Shen-Ju Gou.
